# Using UMAP for Partially Synthetic Healthcare Tabular Data Generation and Validation

**DOI:** 10.3390/s24237843

**Published:** 2024-12-08

**Authors:** Carla Lázaro, Cecilio Angulo

**Affiliations:** 1 Intelligent Data Science and Artificial Intelligence Research Center, Technical University of Catalonia, Nexus II Building, Jordi Girona 29, 08034 Barcelona, Spain; 2 Robotics and Industrial Informatics Institute (CSIC-UPC), Llorens i Artigas 4, 08028 Barcelona, Spain

**Keywords:** physiological sensor data, synthetic data generation, data imputation, smart health, privacy preservation

## Abstract

In healthcare, vast amounts of data are increasingly collected through sensors for smart health applications and patient monitoring or diagnosis. However, such medical data often comprise sensitive patient information, posing challenges regarding data privacy, and are resource-intensive to acquire for significant research purposes. In addition, the common case of lack of information due to technical issues, transcript errors, or differences between descriptors considered in different health centers leads to the need for data imputation and partial data generation techniques. This study introduces a novel methodology for partially synthetic tabular data generation, designed to reduce the reliance on sensor measurements and ensure secure data exchange. Using the UMAP (Uniform Manifold Approximation and Projection) visualization algorithm to transform the original, high-dimensional reference data set into a reduced-dimensional space, we generate and validate synthetic values for incomplete data sets. This approach mitigates the need for extensive sensor readings while addressing data privacy concerns by generating realistic synthetic samples. The proposed method is validated on prostate and breast cancer data sets, showing its effectiveness in completing and augmenting incomplete data sets using fully available references. Furthermore, our results demonstrate superior performance in comparison to state-of-the-art imputation techniques. This work makes a dual contribution by not only proposing an innovative method for synthetic data generation, but also studying and establishing a formal framework to understand and solve synthetic data generation and imputation problems in sensor-driven environments.

## 1. Introduction

Synthetic data generation has emerged as a powerful tool in several domains that struggle with sensitive or scarce tabular data [1,2,3]. In particular, in the health domain, large amounts of data are continuously measured by sensors embedded in medical devices and monitoring systems. However, the sensitive nature of healthcare data and stringent privacy regulations make data sharing and use difficult. Synthetic data can mitigate these challenges by replacing the original data, reducing the risk of private data leakage while still retaining the ability to analyze and train models effectively. Furthermore, synthetic data generation effectively addresses data scarcity, missing data, and a lack of predictors by expanding the data set through artificial means, offering greater volume for analysis and model training.

There are many research areas where data availability presents unique challenges due to stringent privacy concerns, especially in the health domain [4,5,6,7]. These problems stem from the sensitive nature of medical information, such as diagnoses and treatments, which is often included in health records. However, such data are crucial for technological advancements in healthcare, which increasingly rely on accurate and comprehensive data sets. In many healthcare applications, sensor data play a key role in collecting continuous patient information. Therefore, generating synthetic data to augment or replace sensor-based data sets could significantly reduce the reliance on direct measurements, offering a privacy-preserving alternative while maintaining analytical value [8].

Over time, various solutions have been proposed to address privacy risks, with early efforts focusing on anonymization and pseudo-anonymization. These processes entail the removal or encryption of identifiers that link an individual to stored data [9]. While these techniques are beneficial in numerous scenarios, they still present residual privacy risks. Consequently, the generation of synthetic data has been proposed as an alternative or supplementary methodology for the protection of privacy.

As an evolving field, synthetic data generation lacks a unified definition, which has resulted in a variety of interpretations across different contexts [10]. Most definitions agree that synthetic data entail the creation of new data values that mirror the statistical characteristics of the original data set [3,11]. However, a recent working definition, proposed by the Royal Society and The Alan Turing Institute, defines synthetic data as “data that has been generated using a purpose-built mathematical model or algorithm, with the aim of solving a (set of) data science task(s)” [12]. This divergence in definitions is particularly relevant for the validation of synthetic data, where the assessment can be based either on statistical fidelity or the data’s utility in addressing data science challenges.

In [12,13], synthetic data are classified into two principal categories: fully synthetic and partially synthetic. Fully synthetic data sets are entirely artificial and contain no original data. In contrast, partially synthetic data sets replace only certain attributes with synthetic values, resulting in a hybrid data set that combines real and synthetic data [14]. This approach is particularly advantageous for handling sensitive attributes, where synthetic versions can be employed to substitute sensitive values while maintaining the utility of the non-sensitive data.

In cases where the challenge is not related to sensitive data but rather missing values, the focus shifts to data imputation. Data imputation is a widely accepted approach for estimating and filling in missing values based on available data [15]. Given the close relationship between partially synthetic data generation and data imputation, both approaches modify specific attributes while preserving the structure and integrity of the original data set.

The generation of synthetic data in clinical settings primarily involves tabular, time series, and text-based [10,16,17]. This article focuses on the generation of tabular data in the context of healthcare, introducing a novel methodology that leverages data visualization tools to enhance the generation of synthetic data. To the best of our knowledge, no existing methods employ visualization tools to analyze real-world reference data structures for the generation of partially synthetic data.

The goal of this work is to establish a new research direction in partially synthetic data generation. We aim to provide a foundation for the use of dimensionality reduction tools for data synthesis purposes. Ultimately, this line of research aims to extend to visualization-based fully synthetic data and provide a baseline for comparing this approach with more established generative models, including VAEs, GANs, and diffusion models.

This article is structured as follows. Section 2 provides an overview of background information and related work for the generation and imputation of synthetic data, with a particular emphasis on health-related techniques. Section 3 introduces the data sets used for developing the methodology, followed by a comparison of different visualization tools. The section also presents a formal framework to describe the problem and the proposed methodology. Finally, the section outlines the various workflow strategies employed to ensure data privacy. The proposed methodology is tested and validated in Section 4 on different cancer public repositories. Finally, Section 5 reviews the obtained results and discusses their limitations, while Section 6 concludes the article and suggests potential directions for future research.

## 2. Related Work

In this section, the state of the art regarding the topics addressed in the article is reviewed. Specifically, we examine and compare the concepts and methods associated with fully and partially synthetic data, explore various data imputation techniques, discuss data visualization and dimensionality reduction tools, and finally consider approaches to privacy preservation.

### 2.1. Fully and Partially Synthetic Data

The first proposals for the generation of synthetic data are often credited to Refs. [18,19]. These foundational works introduced methodologies based on statistical frameworks and techniques designed to address the issue of missing data. Since these initial proposals, many techniques have been developed to generate synthetic data, with objectives ranging from preventing privacy risks at disclosure to facilitating easier data access for machine learning models. This can involve either partial synthesis, where only some original records are replaced, or full synthesis, where the entire data set is substituted with a synthetic data set of the same size and composition [20].

The distinction between fully and partially synthetic data leads to the differentiation between fully and partially synthetic methods. Fully synthetic methods aim to replicate the statistical properties and relationships inherent in the original data without retaining any direct links to real individuals. While this approach provides robust privacy protection, it may compromise the data fidelity.

Conversely, partially synthetic methods seek to combine real and artificial data [21]. These data sets are typically used when only certain attributes are sensitive, allowing non-sensitive parts to remain unchanged. This approach seeks to balance privacy and data utility. However, partially synthetic data present greater privacy challenges than fully synthetic data [22]. For instance, research in [23] has demonstrated that partially synthetic data are more susceptible to membership inference attacks compared to fully synthetic data, which exhibit greater resilience to such vulnerabilities.

Several techniques have emerged for generating (fully) synthetic data. Among the most prominent techniques are variational autoencoders (VAEs), generative adversarial networks (GANs) and diffusion models (DM), which represent the cutting-edge approaches to synthetic data generation today.

Autoencoder neural networks are composed of two primary components: an encoder and a decoder. The encoder transforms input data into a latent space representation, while the decoder attempts to reconstruct the input from this representation with minimal error. Variational autoencoders (VAEs), introduced in 2013 [24], leverage the autoencoder framework to generate synthetic data.

While VAEs have shown remarkable success in generating images, adapting them for tabular data generation presents unique challenges. To address these challenges, several modifications of the original VAE architecture have been proposed for tabular data generation. One notable approach is Tab-VAE [25], specifically designed to handle the intricacies of tabular data. Further advancements in VAE-based models for tabular data include VAE-GMM [26], which integrates a Bayesian Gaussian mixture model (BGM) within the VAE architecture.

Although VAEs have been explored for synthetic tabular data generation, much of the recent research has focused on generative adversarial networks, first introduced in [27]. GANs consist of two neural networks: the generator and the discriminator, both trained in a competitive setting. The generator is responsible for creating synthetic data that resemble real-world data, while the discriminator’s role is to assess the authenticity of these data.

Although GANs initially found success in synthesizing image data, recent efforts have adapted them for tabular data generation. One notable example is CTGAN [28], which modifies the GAN architecture to handle the specific challenges of tabular data, such as the coexistence of continuous and categorical variables. Another innovative example is PATE-GAN [29], which combines the Private Aggregation of Teacher Ensembles (PATE) framework with GANs to enforce privacy preservation during the data generation process. A recent proposal based on the GAN’s generator–discriminator structure is NextConvGeN [30]. In this model, the generator uses data neighborhoods as input and produces synthetic samples within the convex space of these neighborhoods. The discriminator then evaluates these generated samples by contrasting them against a randomly selected batch of data from the broader data space.

In the healthcare domain, GANs have been applied to generate synthetic patient data. MedGAN [31], for instance, is designed to generate high-dimensional discrete variables, such as binary and count features, by combining an autoencoder with a GAN. This method is especially useful for simulating patient records with mixed data types. CorrGAN [32] builds on the MedGAN architecture by adding a correlation preservation mechanism, ensuring that the generated data maintain the original data attribute correlations. Further improvements to the GAN-based approach for medical data generation were proposed by Camino et al. [33], who introduced the Wasserstein GAN (WGAN) [34] with gradient penalty to stabilize training and improve the quality of the generated data.

Given the wide variety in medical data types, several methods have been proposed for generating multimodal synthetic data. For instance, [35] introduces the Multimodal Conditional GAN (MMCGAN), which combines a conditional GAN for tabular data generation with a model for conditional 3D image synthesis at variable resolutions.

Diffusion models [36,37] are generative models that employ a stochastic process to iteratively transform noise into meaningful data samples. These models have gained significant attention due to their remarkable performance across various generative tasks, particularly in text-to-image synthesis. Unlike GANs, which rely on adversarial training, diffusion models use a progressive denoising process, making them more stable and yielding higher-quality results in many cases, especially for complex data generation.

The impressive success of diffusion models in image synthesis has sparked interest in applying them to the generation of tabular data. One of the most promising examples is TabDDPM [38], a recent diffusion-based model designed specifically for tabular data. Another application of diffusion models can be found in the healthcare sector, where MedDiff [39] was introduced as a generative model for electronic health records (EHRs).

A final noteworthy development is the introduction of a fair diffusion model by Yang et al. [40]. This model is designed to address biases present in sensitive attributes of training data, making it suitable for generating balanced data sets that mitigate issues of fairness.

On the other side, different methods exist for generating partially synthetic data. For instance, a synthesizer algorithm called Perturbed Gibbs Sampler (PeGS) is proposed in [41], which employs a Markov chain Monte Carlo (MCMC) method to generate partially synthetic data.

In addition to classical techniques, machine learning-based methods have also been developed for partially synthetic data. In Ref. [42], a bidirectional recurrent neural network (BiRNN) is proposed to generate partially synthetic biomedical signals. In addition, a long-short-term memory (LSTM) algorithm is utilized in [43] to produce partially synthetic annotated text, which is then used to train a machine learning model for automatic de-identification of protected health information (PHI).

### 2.2. Data Imputation

The practice of data imputation is a commonly employed approach for estimating and filling in missing values within a data set, based on the existing data available for analysis [15]. Over the decades, numerous techniques have been developed to address this problem. Currently, both conventional statistical methods and advanced machine learning- and deep learning-based approaches are employed to tackle data imputation challenges.

Conventional methods encompass mean and median imputation and regression imputation, among others. These methods are classified as single imputation approaches, meaning they do not account for the uncertainty inherent in predicting missing values.

However, multiple imputation (MI), originally proposed in [44], has been widely adopted to handle missing data due to its ability to produce unbiased results [45]. Multiple imputation entails the substitution of missing values in a data set with probable values, imputes, resulting in an imputed data set. This process is repeated multiple times, with slight variations incorporated to the model in each iteration [46], thus producing multiple imputed data sets. Then, the imputed data sets are used for the original data purpose, thereby yielding an individual analysis for each imputed data set. These analyses are then combined to produce an overall analysis [47].

The evolution of models used for obtaining imputes has progressed since the inception of multiple imputation. In recent studies, such as [15,48], different models for data imputation have been compared. These include multivariate imputation by chained equations (MICE), random forest algorithms, denoising autoencoders, and generative adversarial imputation nets (GAINs).

Given that imputed values are also synthetically generated values, there exist significant overlaps between the research domains of data imputation and synthetic data generation. Specifically, the outcome of a data imputation process essentially results in a partially synthetic data set. However, the expression “data imputation” is typically applied in the context of addressing missing data, whereas “synthetic” or “partially synthetic data” are commonly employed either to mitigate concerns about sensitive data or a data augmentation procedure.

Recognizing these similarities and overlap, a pioneering method for generating fully synthetic tabular data sets using imputation techniques was introduced in [49]. This procedure involves an iterative process to remove distinct sets of values, followed by the application of an imputation method to substitute the removed values with new ones. Following multiple iterations, the replaced values from each iteration are combined, resulting in the creation of a comprehensive table containing entirely synthetic data.

### 2.3. Data Visualization and Dimensionality Reduction Tools

The field of data visualization encompasses a range of techniques and tools that facilitate the comprehension of the structural organization and distributional characteristics of complex data sets. The visualization of high-dimensional data presents a significant challenge. To address this issue, data visualization techniques have been developed to reduce data dimensionality from highly dimensional space to a 2D or 3D representation. The most commonly utilized tools are t-distributed stochastic neighbor embedding (t-SNE) [50], Uniform Manifold Approximation and Projection (UMAP) [51], and TriMap [52].

Several studies have explored the relative strengths of these methods for specific applications. For example, in [53] statistical tests were applied to compare t-SNE and UMAP for single-cell data visualization but found no clear advantage of one method over the other. Similarly, Ref. [54] evaluated t-SNE, UMAP, and TriMap, along with other dimensionality reduction techniques, and concluded that each method impacts data sets differently. However, these studies suggest that selecting an appropriate dimensionality reduction method can significantly enhance the outcome.

A significant challenge that is common to all three techniques is the inherent trade-off between the preservation of local and global structures in the data [55]. Although there is no universally accepted definition of local or global structures, the term *local structure preservation* is used to describe the process of maintaining the set of high-dimensional neighbors for each data point in the lower-dimensional space. This encompasses the maintenance of relative distances or rank information among neighboring points, which essentially determines which points are closer in proximity to one another. In contrast, global structure preservation prioritizes the maintenance of relative distances or rank information between more distant points, thereby ensuring the reflection of broader patterns in the reduced dimensional space. In simpler terms, local structure preservation emphasizes maintaining point distances within clusters, while global structure preservation considers the distances between clusters.

A significant limitation of these methods, including t-SNE, UMAP, and TriMap, is the lack of a clear and systematic approach to parameter adjustment for balancing local and global structure preservation. This indicates that users must meticulously select between prioritizing either local or global structures, as it is not possible to optimize for both simultaneously.

Another crucial factor affecting the efficacy of these methodologies is the initialization strategy employed. t-SNE typically utilizes random initialization, UMAP applies a technique called Laplacian eigenmaps [56] and TriMap commences with principal component analysis (PCA). However, both UMAP and TriMap can be initialized randomly, as documented in the original papers [51,52]. Despite the tuning of hyperparameters, random initialization frequently results in suboptimal outcomes for both UMAP and TriMap [55,57].

Data visualization tools have demonstrated their utility in various health-related studies, particularly with UMAP. For instance, UMAP is used in [58] to visualize electronic health records (EHRs) from an emergency department, aiming to identify patient populations similar to a specific patient and provide additional information for the triage process. Similarly, UMAP is employed in [59] to extract patient features for detecting depression from data generated by wearable IoMT (Internet of Medical Things) devices. Furthermore, a UMAP-based methodology is proposed in [60] to identify minimal residual disease (MRD), a strong prognostic factor in two different types of leukemia.

UMAP has also shown significant capabilities in the efficient management and reduction of very high dimensional data. For example, in [61], UMAP is applied to five data sets with varying, high-dimensional structures: MNIST [62] (784 features), Fashion MNIST [63] (784 features), USPS [64] (256 features), Pen Digits [65] (64 features), and UMIST Face Cropped [66] (10304 features). Similarly, it is introduced in [67] a successful reduction from 6170 dimensions—representing genes in each of 1484 single-gene deletion strains—to a 2D space using UMAP. These studies showcase UMAP’s adaptability in transforming high-dimensional data into meaningful, low-dimensional representations without significant information loss.

On the other side, dimensionality reduction has also demonstrated significant value for imputation purposes. In [68], a novel technique was proposed to impute medical data records by applying a feature reduction method by class-based clustering. Another method, UMAP-SMOTENC [69] combines UMAP with the SMOTE-NC oversampling technique to work in a non-linear, compressed version of the data space. This approach effectively generates synthetic, privacy-preserving data while preserving essential patterns from the original data set, particularly those necessary for accurate classification tasks.

### 2.4. Privacy Preservation

Given the extensive use of healthcare data when developing algorithms for data synthetic generation and data imputation, several privacy threats have been identified in [70]: (i) identity disclosure or reidentification, where an adversary reveals the identity of a patient; (ii) attribute disclosure, where an adversary uncovers sensitive attributes of a patient; and (iii) membership disclosure, where an adversary successfully infers the presence of a patient in published data.

Ensuring privacy in the exchange of medical data among practitioners while protecting sensitive information has emerged as a critical challenge. Additionally, the monetary value of health data makes it a common target for attacks [22]. While synthetic data can mitigate some privacy issues by avoiding explicit one-to-one matches with real individuals, it can still inadvertently leak information related to training samples [23]. To address this risk, methods such as introducing statistical perturbation through differential privacy have been proposed [71].

## 3. Materials and Methods

In this section, the key components for developing the proposed methodology are presented. First, the data sets used in this study are introduced. This is followed by an evaluation of the most appropriate visualization method for implementing the methodology. The section then outlines the proposed methodology, starting with the foundational concepts, followed by the detailed proposal for partially synthetic data generation. Finally, strategies for ensuring data privacy and workflow management between different data centers are discussed to address privacy concerns and facilitate secure data exchange.

### 3.1. Datasets

The proposed methodology is designed to be applicable to various topics within the health domain. In this case, the focus is specifically on prostate cancer and breast cancer data.

#### 3.1.1. Prostate Cancer

In the case of prostate cancer, two public data sets are utilized: the Prostate Imaging and Cancer Analysis Information (PI-CAI) dataset, available at https://pi-cai.grand-challenge.org/DATA/ (accessed on 7 March 2024) and the Cancer Imaging Archive (CIA) dataset available at https://www.cancerimagingarchive.net/collection/prostate-mri-us-biopsy/ (accessed on 19 March 2024).

The PI-CAI data set comprises 1500 samples, each containing 12 features related to prostate cancer. The attribute case_csPCA is the primary outcome variable, indicating the presence of clinically significant prostate cancer. Certain features, such as patient_id, study_id, and mri_date, are excluded from this analysis due to their irrelevance for predicting case_csPCA and the potential privacy concerns they pose. Additionally, the attribute histopath_type, which refers to the procedure used to sample lesion tissue, is disregarded in this study because it does not directly impact the diagnosis of prostate cancer.

The attribute lesion_GS represents the Gleason Score (GS), a grading system that evaluates the aggressiveness of prostate cancer based on the microscopic appearance of cancer cells. Another important feature, lesion_ISUP, simplifies the Gleason Score into five distinct ISUP grades to better predict clinical outcomes. Furthermore, case_ISUP denotes the highest ISUP grade among the sampled lesions. Additional features include psa, which represents the prostate-specific antigen level, and psad, which is the prostate-specific antigen density. In cases where psad is missing, it can be computed by dividing PSA by the prostate volume (psad_computed).

Since case_ISUP reflects the highest risk grade in a sample, initially it is considered instead of lesion_ISUP. However, a correlation analysis revealed a substantial correlation coefficient (0.87) between case_ISUP and case_csPCA, leading to the exclusion of case_ISUP as a predictor. Consequently, patient_age, prostate_volume, and either psa, psad, or psad_computed are selected as predictors for the output case_csPCA.

The CIA data set, also a public repository, provides a wide range of information on prostate cancer. However, unlike the PI-CAI data set, the case_csPCA attribute is not available. Despite this limitation, the data set contains psa and prostate_volume values for each of its 24,783 samples. The attribute patient_age, however, is missing. Given the size and diversity of the CIA data set, it offers a valuable resource for exploring correlations and patterns in prostate cancer. The objective is to generate synthetic values for patient_age and assign synthetic case_csPCA values to each sample based on the available information.

#### 3.1.2. Breast Cancer

This study utilizes two publicly available breast cancer datasets. The first dataset, referred to as BC-MLR, is the Breast Cancer dataset from the UCI (University of Chicago, Illinois) Machine Learning Repository, available at https://archive.ics.uci.edu/dataset/14/breast+cancer (accessed on 10 July 2024). The second dataset, BCNB, is the Early Breast Cancer Core-Needle Biopsy WSI dataset, accessible at https://paperswithcode.com/dataset/bcdalnmp (accessed on 8 July 2024).

The BC-MLR data set comprises 286 instances with nine attributes related to patient characteristics and tumor details. Patient-specific data include age at the time of diagnosis, represented by the age attribute, as well as the patient menopausal status at diagnosis, noted in the menopause feature. Tumor-related information is captured through variables such as tumor size, which records the maximum diameter of the excised tumor, and deg-malig, reflecting the tumor degree of malignancy and aggressiveness.

Additional features in the BC-MLR data set address the extent of cancer spread, with inv-nodes indicating the number of lymph nodes involved in metastatic breast cancer, and node-caps providing information on whether the tumor has replaced the lymph and penetrated the capsule. Moreover, the data set records the affected breast side (breast) and the specific breast quadrant where the tumor is located (breast-quad). The data set also includes irradiat, which specifies whether the patient has undergone radiation therapy. The target variable in this data set is Class, which represents whether there is cancer recurrence after treatment.

On the other hand, the BCNB data set includes information from 1058 breast cancer patients. The age attribute records the patient age at the time of diagnosis, while tumor characteristics such as tumor size and tumor type provide detailed descriptions of the tumor. Additionally, the data set captures molecular markers such as the expression of estrogen receptor (ER), progesterone receptor (PR), and human epidermal growth factor receptor 2 (HER2), represented by the features ER, PR, and HER2, respectively. The data set also includes HER2 expression, which provides the results of an immunohistochemistry (IHC) test, used to predict how well the cancer will respond to treatment.

The BCNB data set further includes the histological grading feature, which assesses how closely the tumor cells resemble normal breast cells. Information regarding surgical interventions, specifically the type of surgery performed to remove nearby lymph nodes, is contained in the surgical feature. Other features of note include the Ki67 score, which measures the rate at which cancer cells are dividing, and the molecular subtype, a classification system grouping breast cancers based on shared characteristics and hormone receptor status. Finally, the data set provides information on lymph node involvement through features such as the number of lymph node metastases and ALN status, indicating whether the lymph nodes in the underarm area are affected.

Since ideally the database would have the same features, we focus on common features between the data sets. Therefore, particular attention is given to the age attribute, tumor size, and number of lymph node metastases, as these variables are present in both the BC-MLR and BCNB data sets.

### 3.2. Comparison of Visualization Tools for Database Representation

As discussed in Section 2.3, the most commonly utilized tools for 2D visualization of high-dimensional data include t-SNE, UMAP, and TriMap. Each of these methods has its advantages and limitations, particularly in their ability to preserve local and global structures.

In the context of this methodology, reproducibility and effective cluster visualization are of paramount importance. Specifically, we are interested in distinguishing clinically significant prostate cancer samples within the PI-CAI data set and cancer recurrence cases in the BC-MLR data set. Therefore, all three methods—t-SNE, UMAP, and TriMap—were applied to visualize the most comprehensive data set available for the two types of cancer, PI-CAI and BC-MLR, after normalizing the data to ensure consistency in the results.

**Figure 1:**
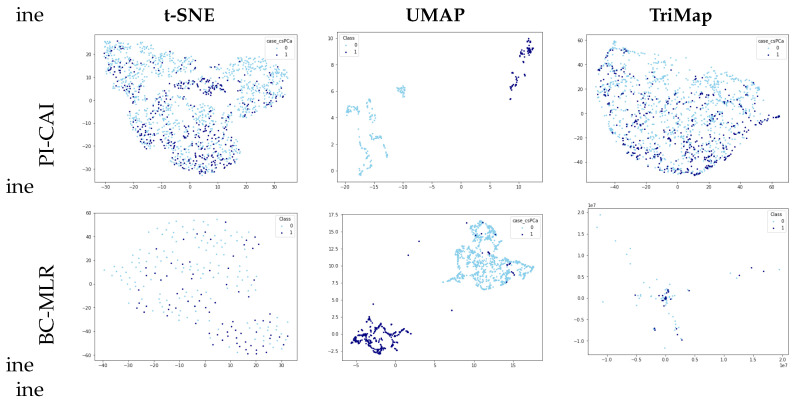
PI-CAI and BC-MLR visualizations using different dimensionality reduction techniques.

 Figure 1 presents the visualizations of the PI-CAI and BC-MLR data set using t-SNE, UMAP, and TriMap. Upon examination, it is evident that UMAP offers a more distinct separation of classes compared to the other methods. Neither t-SNE nor TriMap displays clear differentiation between the classes, highlighting UMAP’s superior ability to organize the data meaningfully.

Based on these findings, this study focuses on UMAP as a dimensionality reduction tool. Aside from the observed capacity to effectively organize data in the form of clusters, it presents high reproducibility [72].

By default, UMAP embeds data into Euclidean space, typically in a 2D plane, which allows for clear and intuitive interpretations. Although UMAP allows for other embedding spaces, Euclidean space is specifically chosen for this study, as it facilitates the use of Euclidean distance for measuring similarities between data representations. Additionally, given the nature of medical data, periodic or other complex behaviors that would warrant the use of non-Euclidean spaces are not expected. The Euclidean space provides a more intuitive and appropriate framework for capturing the relationships inherent in medical data sets.

### 3.3. Proposed Methodology

The proposed procedure is designed to facilitate the exchange of medical data, with the primary goal of augmenting or completing data sets for use in data-driven solutions. Let us imagine hospital 1 with a private, complete, and small data set. On the other side there is hospital 2, with a larger data set, but in this case uncompleted. Our methodology allows hospital 2 to leverage the complete information in hospital 1 without sharing the actual data.

The methodology begins by extracting information from the complete data set in hospital 1, which is named the reference data, and transforming it into a two-dimensional space using UMAP. In a second step, using this transformed data map, along with the trained UMAP function, synthetic values are assigned to the incomplete data set (from hospital 2). These newly assigned synthetic values are then mapped back into the 2D representational space to evaluate their congruence with the reference data.

**Figure 2:**
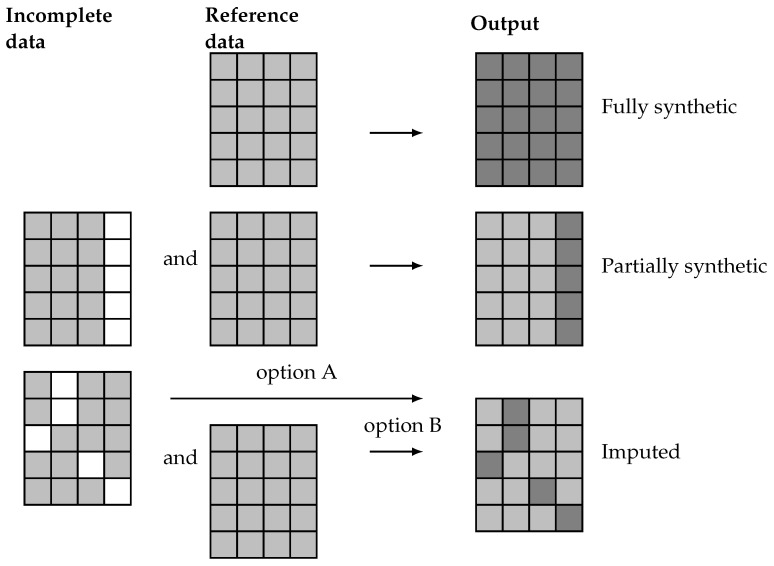
Representation of real (light gray) and artificial (dark gray) values for missing data (white) on fully synthetic, partially synthetic, and imputed data sets.

The proposed methodology is a general procedure able to generate artificial data which, as previously discussed, can be categorized into fully synthetic, partially synthetic, and imputed data. These different results are achieved trough different methods, as illustrated in Figure 2. There, it is highlighted how fully synthetic methods generate data sets that contain no real data from the reference or incomplete data sets. In contrast, partially synthetic methods produce data sets that combine real data from an incomplete data set with artificial data generated from the available information. Finally, imputation methods, which typically address missing data, result in data sets that contain a mix of real and artificial data. These imputation methods can either generate the missing values from only the actual incomplete data set (option A) or complement it with a reference data set containing real-world data (option B).

Since partially synthetic data aim to protect sensitive information and imputed data seeks to resolve missing data issues, the artificial data in the resulting data sets usually exhibit different distributions. Partially synthetic data typically include some synthetic features that are consistent across all entries. Conversely, the artificial values in an imputed data set do not necessarily correspond to the same feature for all entries.

The relevant elements involved in the definition of each type of problem and the steps in the proposed methodology are denoted as follows.

### 3.4. Formal Framework

In this subsection, we aim to provide a formal definition of the proposed framework for synthetic data generation. The following subsections introduce the real and synthetic datasets, formally define the problems addressed, establish their equivalence, and explore the role of visualization tools in the methodology.

#### 3.4.1. Real-World Data Sets

Let $D_{RW}=\left(X,Y\right)={\left\{\left(x_{i},y_{i}\right)\right\}}_{i=1}^{N}$ be a real-world data set, with *N* instances composed of *d* features $x=(x^{\left(1\right)},\ldots ,x^{\left(d\right)})$ and $d_{y}$ outputs $y=(y^{\left(1\right)},\ldots ,y^{\left(d_{y}\right)})$. Values for some features can be unknown for all the *N* instances in the data set. Considering that known and unknown features are ordered, instances can be noted:

(1) $$x_{i}=\left(x_{i}^{K},x_{i}^{U}\right)=\left(\left(x_{i}^{\left(1\right)},\ldots ,x_{i}^{\left(d_{K}\right)}\right),\left(x_{i}^{\left(d_{K}+1\right)},\ldots ,x_{i}^{\left(d\right)}\right)\right)$$

with $x_{i}^{K}$ and $x_{i}^{U}$ representing known (light gray cells in Figure 2) and unknown (white cells in the same figure) values for all the instances, respectively. It is important to note that unknown features may not necessarily be absent from the data set but are considered unknown due to privacy concerns, necessitating the generation of synthetic values for these sensitive features. Hence, the real-world data sets considered in the problem can be represented as

(2) $$D_{RW}=\left(X^{K},X^{U},Y\right)=\left\{\left(x_{i}^{K},x_{i}^{U}\right),y_{i}\right){)\}}_{i=1}^{N}$$

with $\dim\left(X^{K}\right)=d^{K}$, $\dim\left(X^{U}\right)=d^{U}$, and $\dim\left(Y\right)=d_{y}$. As discussed previously, two types of real-world data set are distinguished in this context: a reference data set and an incomplete data set. For the reference data set, $D_{r}$, all values are known for all the features, represented by $d^{K}=d$. Conversely, in the incomplete data set, $D_{i}$, values for at least one feature are missing, $d^{U}=d-d^{K}\geq 1$. Note that in both cases the output Y can be known, unknown, or partially known.

#### 3.4.2. Synthetic Data Sets

Let $D_{g}$ denote a synthetic data set (output data set), with ${\hat{x}}_{i}^{U}$ denoting the synthetic values generated in some form for the unknown features of the *i*-th entry. Depending on the employed methodology for its generation, the output database can be fully synthetic,

(3) $$D_{g}=\left({\hat{X}}^{U},\hat{Y}\right)=\left\{{\hat{x}}_{i}^{U},{\hat{y}}_{i}\right){\}}_{i=1}^{N}$$

or partially synthetic,

(4) $$D_{g}=\left(X^{K},{\hat{X}}^{U},Y\right)=\left\{\left(x_{i}^{K},{\hat{x}}_{i}^{U}\right),y_{i}\right){\}}_{i=1}^{N}$$

Note that in the case of fully synthetic data, the output Y must be necessarily generated ($\hat{Y}$) because no incomplete data are used in the generation process. Conversely, for partially synthetic data, the output can either be real, denoted as $D_{g}=\left(X^{K},{\hat{X}}^{U},Y\right)$, or synthetic, $D_{g}=\left(X^{K},{\hat{X}}^{U},\hat{Y}\right)$, in cases where the output is unknown in the incomplete data and must therefore be generated. Both cases are considered later in the detailed description of the methodology.

#### 3.4.3. Problem Definition

According to the provided notation, the three particular cases of the general synthetic data set generation problem can be defined as follows:

1. **Fully synthetic generation.** The proposed methodology aims to generate a fully synthetic data set, $D_{g}$, based on a real-world reference data set, $D_{r}$, with $N_{r}$ instances and *d* features.
2. **Partially synthetic generation.** This approach also relies on a reference data set, $D_{r}$, with $N_{r}$ instances and *d* features, along with an incomplete database with missing features. For simplicity, in this study we assume that dim(Y)=1 and only one feature (*x*) is missing in the incomplete data set, $\dim(X^{U})=1$, and this is also the only feature to be synthetically generated. The incomplete data set is denoted as $D_{i}=D_{r-\{x\}}$, which has $N_{i}$ instances. Thus, there are d−1 known features and one unknown feature common to all instances. The goal is to generate a partially synthetic data set, $D_{g}$, which includes known features and synthetic values for one unknown feature.
3. **Imputation.** Imputation methodologies operate on an incomplete database with missing features, similar to the previous case. However, missing features may vary across entries. For instance, considering one missing feature for each entry, the incomplete data set is defined as $D_{i}=D_{r-\{x_{j}\}}={\left\{\left(x_{i}^{K},x_{i}^{U}\right),y_{i}\right\}}_{i=1}^{N_{i}}-\left\{x_{j}\right\},j\in \left\{1,2,\ldots ,d\right\}$. In this case, the imputation can rely on the non-missing values of the incomplete database (option A) or on a reference data set with no missing values (option B).

#### 3.4.4. Problem Equivalence

**Figure 3:**
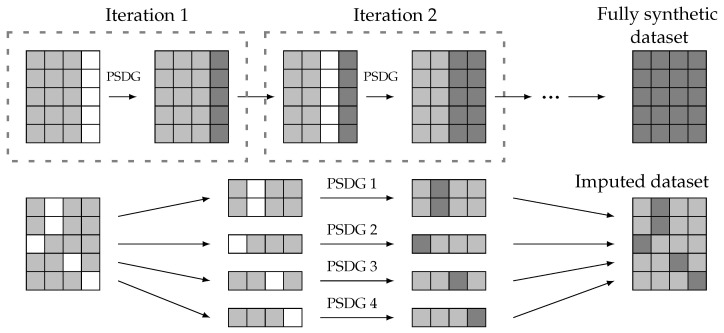
Fully synthetic and imputed data generation from partially synthetic data generation (PSDG) methods. Real values (light gray), artificial values (dark gray), and missing data (white).

A viable approach to generating fully synthetic data starts with the same initial setup as partially synthetic data generation: a reference data set $D_{r}$ and an incomplete data set $D_{i}$. In this scenario, all features of the generated data should be synthetic. Therefore, the method for partially synthetic data generation (PSDG) can be iteratively applied, each time addressing a different missing feature until the generated data encompass all features. This approach is illustrated in Figure 3 (upper side), where each feature is synthesized in a separate iteration until a fully synthetic data set is obtained.

In addition, imputation methods can be considered a specific case of partially synthetic data generation. Both approaches share a common initial structure with known and unknown features; however, the missing features in an imputation setup are not uniform across all entries. Consequently, the data set with missing values can be divided into multiple subsets, and imputation can be performed using partially synthetic methods, as depicted in Figure 3 (lower side).

Since both fully synthetic methods and imputation methods can be derived from a partially synthetic method, this paper focuses on developing a methodology for partially synthetic data. The proposed approach can be extended to the other two cases in a straightforward form.

#### 3.4.5. Visualization Tools

Finally, we propose the use of visualization tools as a valid methodology for PSDG. Therefore, some notation is introduced. Let $f_{umap,RW}:X⟶C_{RW}$ define a UMAP function trained with real-world data $D_{RW}$, projecting the *d*-dimensional data input space X to the latent space of 2D coordinates, $C_{RW}$. Depending on the origin of the high-dimensional data, the resulting coordinates are denoted as $C_{r}=f_{umap,r}\left(X\right)$, when the map corresponds to the reference data, or $C_{g}=f_{umap,r}\left(\hat{X}\right)$ for the (fully, partially) synthetically generated data set. Note that for both cases, the UMAP function is trained on the reference data set, $D_{r}$.

Let $D_{v}$ be the valid synthetic data, corresponding to the generated samples that are suitable and align well with the reference data, thus $D_{v}\subset D_{g}$. Specifically, the valid synthetic coordinates, $C_{v}\subset C_{g}$, are deemed valid when they share a common underlying structure with the reference coordinates, $C_{r}$, in the 2D latent space:

(5) $$C_{v}=f_{umap,r}\left(\hat{X}\right)\;∼\;C_{r}=f_{umap,r}\left(X\right)$$

### 3.5. Partially Synthetic Methodology Proposal

**Figure 4:**
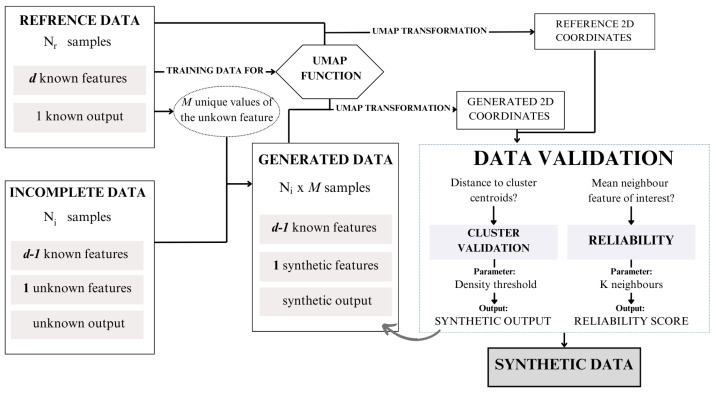
Synthetic data generation framework proposal.

The proposed methodology aims to integrate both real-world reference data and incomplete data to generate a complete partially synthetic data set. This approach leverages a structured framework to ensure both data utility and privacy preservation. The overall workflow of the methodology is illustrated in Figure 4, providing a visual representation of the process steps and their interconnections.

To operationalize this framework, we have developed an algorithm, presented as Algorithm 1. This algorithm outlines the detailed steps required to execute the methodology, including preprocessing, synthetic data generation, validation, and final data set compilation. In this section, the different steps presented in the algorithm are described.

As previously described, the initial setup consists of a reference database, $D_{r}$, and an incomplete database, which contains the same features as the reference data, except for the missing feature *x*, $D_{i}=D_{r-\{x\}}$.

**Step 1: Preprocessing and setup**

The first step, before applying the methodology, is to ensure uniformity in data structures between the incomplete and reference databases. This setup involves aligning units, sampling groups, data collection methodologies, and normalization techniques.

Let us assume that, from $D_{r}$, the range *M* of finite possible values for the feature *x* can be obtained. For categorical, logical, or ordinal variables, this process is straightforward. However, if the missing variable is continuous, a quantization preprocess is applied, which discretizes the continuous values into a finite set of intervals or levels.

**Step 2: Synthetic data generation**

Using the *M* possible values of the missing feature *x*, *M* different entries can be created for each sample out of $N_{i}$ in $D_{i}$ by assigning all different values of the range to *x*. Consequently, a resulting generated database, $D_{g}=D_{r-\left\{x\right\}+\left\{\hat{x}\right\}}$, is obtained, where $\hat{x}$ is the values assigned to the missing feature. Considering that the incomplete database, $D_{r-\{x\}}$, contains $N_{i}$ samples, $D_{g}$ contains $N_{i}\cdot M$ samples and *d* features (d−1 of these are real-world features and one feature is synthetically generated).

**Step 3: UMAP transformation**

The core of the proposed methodology lies in utilizing the visualization tool UMAP to transform data from *d* dimensions to coordinates in two dimensions. The UMAP function is trained with $D_{r}$, resulting in $f_{umap,r}$. This function enables the transformation of not only $D_{r}$, but can also be applied to $D_{g}$. By performing these transformations, the UMAP coordinates of the generated data, denoted as $C_{g}$, can be obtained and compared to the coordinates of the reference data, $C_{r}$.

**Step 4: Reliability score assignment**

Now, a validation step, defined as the reliability score assignment, determines which of the generated samples $D_{v}\subset D_{g}$ are suitable and which do not align well with the reference data. The comparison between samples involves calculating the mean value of the *x* feature for the *k*-nearest neighbors in $C_{r}$ for the 2D coordinates in $C_{g}$. The mean value for the *k* neighbors around the *i*-th sample is denoted as ${\bar{x}}_{i,k}$. Validation entails comparing the $\hat{x}$ value of instance *i* (${\hat{x}}_{i}$) in $D_{g}$ with ${\bar{x}}_{i,k}$. Depending on the feature disparity, defined as the Euclidean distance $f_{\mathrm{disp}}=\left|\right|{\hat{x}}_{i}-{\bar{x}}_{i,k}\left|\right|$, the generated *i*-th sample obtains a certain reliability score, as outlined in Table 1.

If the reliability score $r_{c}$ of a certain sample exceeds a threshold, then the generated *i*-th sample is validated. For a single original sample in the incomplete database, $D_{r-\{x\}}$, there may be multiple suitable values for *x*, resulting in the generation of additional samples. Therefore, the methodology not only imputes the real missing value, but also creates additional suitable samples, which constitute synthetic data.

**Step 5: Cluster validation (if applicable)**

For the last step, we can consider that the output is also unknown $D_{r-\{x,y\}}$. Since the output is known for the reference data, instances can be grouped into clusters, so the output of the $N_{i}$ entries can be assigned based on the distance between each generated coordinate $C_{g}$ and the cluster’s centroid coordinates of $C_{r}$. This process, known as cluster validation, involves defining a radius around each centroid until the density of the cluster samples within the radius falls below a predefined threshold. All samples from $C_{g}$ falling within these cluster areas are then assigned to the corresponding cluster $D_{g}=D_{r-\left\{x,y\right\}+\left\{\hat{x},\hat{y}\right\}}$. Conversely, samples lying outside any cluster area do not meet the criteria for cluster validation.

Adjusting parameters such as the sample density threshold for cluster validation, the number of neighbors considered for reliability score assignment (*k*),and the minimum reliability score for validated samples can significantly affect the outcome of the synthetic data generation process. These parameters should be carefully fine-tuned according to the specific objectives and requirements of the generated data.

Setting a minimum reliability score threshold for validated samples ensures that only high-quality synthetic data points are retained for downstream applications. This threshold serves as a filter to enhance the reliability and usefulness of the generated data.

Furthermore, the sample density threshold determines the extent of cluster validation by defining the area around each cluster centroid. By adjusting this threshold, one can control the inclusion of the cluster validation process. Similarly, varying the number of neighbors (*k*) considered for the reliability assessment of characteristics allows flexibility in evaluating the consistency of feature values among neighboring samples.

**Step 6: Output**

Upon completing the reliability score analysis and, if applicable, the cluster validation process, the algorithm produces a final set of synthetic samples. These samples are selected based on meeting the reliability score threshold, ensuring their consistency with the reference data. Additionally, for scenarios where the target variable is unknown, the algorithm assigns a predicted target value through the cluster validation process. The resulting data set consists of high-quality synthetic samples that are suitable for downstream applications.
**Algorithm 1** Synthetic Data Generation with UMAP-based Dimensionality Reduction**Require:** Complete reference dataset $D_{r}$, incomplete dataset $D_{i}$**Ensure:** Validated synthetic dataset $D_{v}$  1:**Step 1: Preprocessing and Setup**  2:Align data structures: units, normalization, and sampling techniques.  3:Define range of possible values *M* for the missing feature *x* in $D_{i}$.  4:**if** *x* is continuous **then**  5:    Apply quantization to obtain discrete intervals for *M*.  6:**end if**  7:**Step 2: Synthetic Data Generation**  8:Create a *generated dataset* $D_{g}$ by assigning all *M* values to the missing feature *x* for each sample in $D_{i}$.  9:Size of $D_{g}$: $|D_{g}\left|=\right|D_{i}\left|\times |M\right|$.10:**Step 3: UMAP Transformation**11:Train UMAP model $f_{\mathrm{umap},r}$ on $D_{r}$ to obtain 2D coordinates $C_{r}$.12:Apply $f_{\mathrm{umap},r}$ to $D_{g}$ to obtain 2D coordinates $C_{g}$.13:**Step 4: Reliability Score Assignment**14:**for** each sample *i* in $C_{g}$ **do**15:    Find *k* nearest neighbors in $C_{r}$.16:    Compute mean value ${\bar{x}}_{i,k}$ for the missing feature from *k* neighbors.17:    Calculate feature disparity $f_{\mathrm{disp}}=\left|\right|{\hat{x}}_{i}-{\bar{x}}_{i,k}\left|\right|$.18:    Assign reliability score $r_{c}$ based on $f_{\mathrm{disp}}$ using Table 1.19:    **if** $r_{c}\geq r_{\min}$ **then**20:        Validate sample *i* and add to $D_{v}$.21:    **end if**22:**end for**23:**Step 5: Cluster Validation (if applicable)**24:**if** output *y* is also missing **then**25:    Group reference data $C_{r}$ into clusters.26:    **for** each sample *j* in $C_{g}$ **do**27:        **if** *j* lies within cluster radius **then**28:           Assign cluster label and add to $D_{v}$.29:        **end if**30:    **end for**31:**end if**32:**Step 6: Output**33:Return the validated synthetic dataset $D_{v}$.

### 3.6. Data Privacy and Workflow Strategies

The proposed methodology is designed not only to augment or complete data sets, but also to ensure data privacy when the reference and incomplete data sets are located in different data centers. While the previous section detailed the methodology for general reference and incomplete data sets, this section focuses on the information workflow between separate data centers, emphasizing the protection of sensitive information.

To illustrate this, we revisit the scenario involving hospital 1 and hospital 2. In this case, hospital 2 ($H_{2}$) possesses a large but incomplete data set and seeks to leverage the complete data owned by hospital 1 ($H_{1}$) to fill in the missing information. Our methodology offers a solution to this challenge by enabling data exchange without compromising privacy, ensuring that sensitive data remain secure throughout the process.

Three scenarios are considered depending on the unknown information in the incomplete data set in $H_{2}$:

- Case 0: The output is unknown, but the features are all known. In the case of missing outputs or classes, the validation process involves cluster validation, which requires the centroid cluster coordinates of $D_{r}$ and a threshold radius from $H_{1}$.
- Case 1: The output is known, but there is one missing feature. If the interest lies in assigning generated values for a missing feature, the validation process involves a reliability score assignment. This requires the mean value of the feature *x* for the *k*-nearest neighbors in $C_{r}$, for each instance *i* in $C_{g}$, ${\bar{x}}_{i,k}$.
- Case 2: Both, the output and a feature are unknown. For scenarios involving the generation of both the unknown feature and the assignment of sample clusters, the validation process includes both steps.

Regardless of the scenario, all workflows require the UMAP function trained with the reference data in $H_{1}$.

Our primary interest lies in case 1, which results in a synthetic feature for each entry. The other cases are presented for a better understanding of the system, but the workflow proposal focuses on case 1.

**Figure 5:**
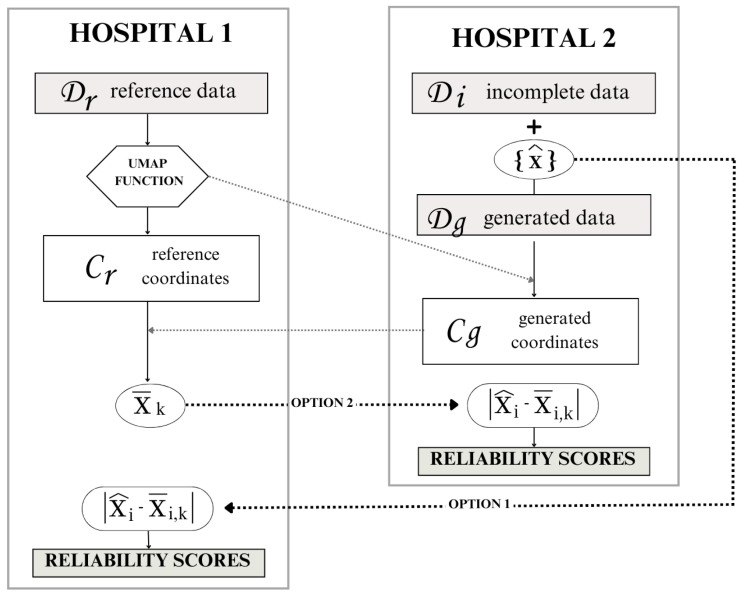
Workflow options according to data privacy.

Depending on the interests and needs of the health institutions and their data centers, there exist different workflow options for sharing information, shown in Figure 5. The UMAP function, $f_{umap,r}$, trained with the data in $H_{1}$, must be shared with hospital 2. This step does not pose any privacy risks because the reference data cannot be obtained back from the trained function.

The second information exchange occurs when hospital 2 transmits the UMAP coordinates, $C_{g}$, to the reference institution, hospital 1. Notably, in this exchange, $H_{1}$ does not obtain sensitive high-dimensional data due to the inherent limitations of UMAP coordinates. Since both the UMAP transformation, and its inverse, are stochastic processes, noise inevitably accompanies every transformation. Consequently, $H_{1}$ can only approximate the generated data from $H_{2}$ by inversely transforming the shared coordinates $C_{g}$. The information exchange in this step is crucial for computing the mean value of the feature *x* for the *k* neighbors in the reference coordinates for each generated sample.

Finally, to perform the reliability score assignment, there are two options:

- **Option 1:** Hospital 2 shares the generated value ${\hat{x}}_{i}$ for each sample with hospital 1. Hospital 1 calculates the feature disparity $f_{\mathrm{disp}}=\left|{\hat{x}}_{i}-{\bar{x}}_{i,k}\right|$ and assigns a reliability score $r_{c}$ to each generated sample. Hospital 1 then sends the reliability scores back to hospital 2 for them to keep the desired samples.
- **Option 2:** Hospital 1 shares the mean neighbor value of feature *x* for each sample *i* with hospital 2. Hospital 2 calculates the feature disparity to provide the reliability scores.

## 4. Results

In this section, the proposed methodology is applied to generate synthetic data using the data sets introduced in Section 3.1. Since the true values for the missing data in the incomplete data sets are not available, direct validation of the generated synthetic data cannot be performed. As an alternative, a validation process is carried out using the PI-CAI data set. This involves partitioning PI-CAI into reference and incomplete data sets, allowing the generated synthetic values to be compared against the original values. This comparison facilitates a thorough evaluation of the performance and accuracy of the proposed methodology.

### 4.1. CIA Synthetic Data Generation from PI-CAI

In our experimental setup, we aim to generate synthetic samples for the CIA database using the PI-CAI data as a reference. Therefore, the reference data set comprises three features and a target variable per sample and the incomplete data set lacks one feature and the target variable.

Of particular importance is the consistency of features and units across databases. Notably, PSA (prostate-specific antigen) measurements in both the CIA and PI-CAI data sets are recorded in ng/mL, while prostate volume is documented in cc (cubic centimeters) for CIA and mL (milliliters) for PI-CAI, both equivalent units.

Before applying the UMAP transformation to the reference data, a data normalization is conducted to enhance visualization, specifically focusing on the age distribution in the UMAP plot, which is crucial for subsequent data validation. The chosen normalization method is Standard Scaler, as it yields the most favorable distribution post-UMAP transformation compared to other normalization techniques, especially regarding outliers.

After applying normalization and UMAP transformation to the reference data set, we ascertain the 2D coordinates of the PI-CAI reference samples. The resulting distribution has already been illustrated in  Figure 1. As can be observed, aside from some outliers, the output variable facilitates the definition of clusters.

The patient_age feature in the reference database shows values from 35 to 92, therefore the number of possible values for the feature of interest is M=58. By assigning to each sample on the incomplete database all the possibles ages, the resulting synthetic data set is composed of 58×24,728=1,434,224 potential entries.

**Figure 6:**
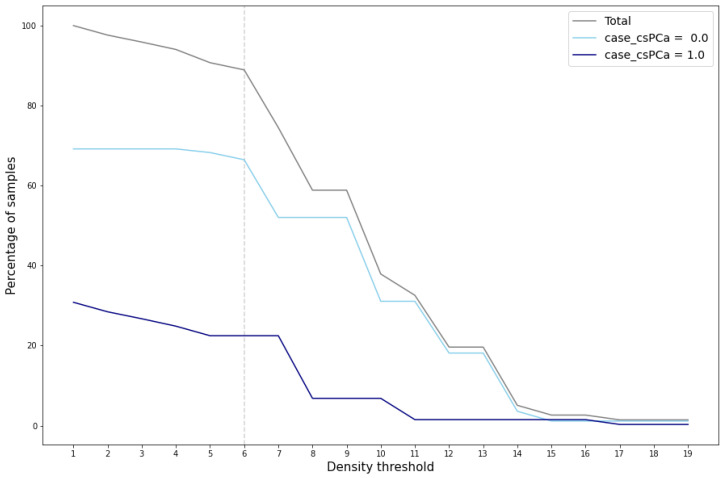
Percentage of cluster-validated samples for different density thresholds.

The evaluation and validation of the generated data reveal several key findings. Cluster validation depends on the density threshold utilized to define the cluster area. Figure 6 illustrates the percentage of total generated samples that achieve cluster validation across various density thresholds, segmented by cluster and total count.

A marked reduction in the number of validated samples, particularly within the cluster case_csPCA=0, is evident when the density threshold exceeds the value of 6. This significant decrease is also reflected in the total percentage of validated samples, which notably drops at this threshold value. This reduction indicates that for density thresholds greater than 6, the area around the cluster case_csPCA=0 centroid fails to encompass the entire cluster. For the cluster case_csPCA=1, this phenomenon occurs at a density threshold of 7.

**Figure 7:**
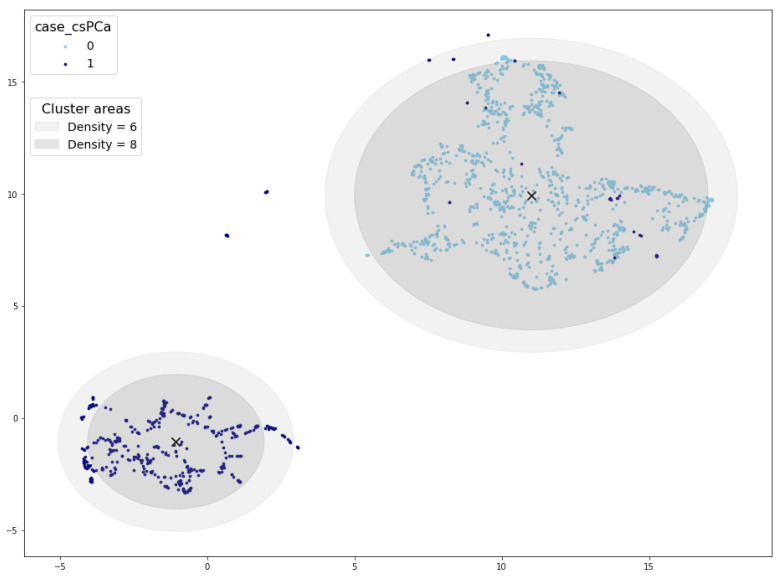
Cluster areas for density threshold equal to 10 (inner circle) and 8 (outer circle). Cluster centroid marked with a black ×.

Examining Figure 7, which depicts the cluster area for density thresholds set at 6 and 8, further elucidates the disparity in validated samples. It becomes evident that density values exceeding 6 result in a radius smaller than the cluster radius, consequently leaving some samples outside the cluster area. In such cases, synthetic samples situated at the periphery of the cluster would not pass the cluster validation process.

**Figure 8:**
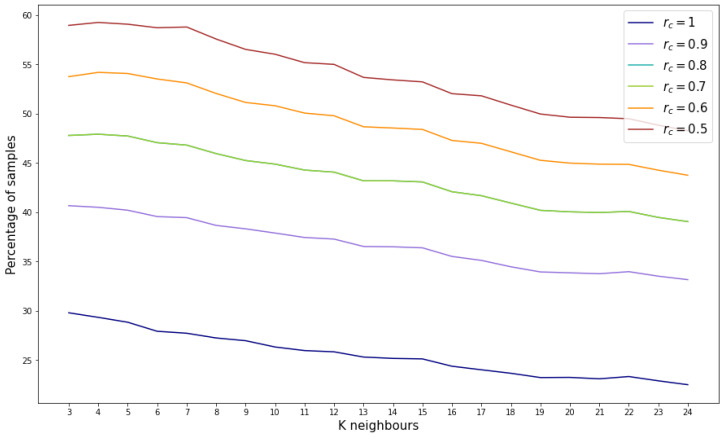
Percentage of samples by reliability score for different number of considered neighbors.

Based on the aforementioned findings, the density threshold for this reference database is set to 6. Moving forward to the reliability score assignment, the outcomes depend on the selected number of neighbors (*k*) used to compute the mean age. It is also important to adjust the ϵ value for the concrete feature generation, in this case set to 1. Figure 8 illustrates the variation in the percentage of samples assigned specific reliability scores as *k* increases. Notably, there is a slight decrease in the percentage of samples as *k* increases. Conversely, as the reliability score decreases, there is a corresponding increase in the percentage of samples.

However, considering the most stringent reliability score ($r_{c}=1$), the percentage of validated samples ranges between 20% and 30%, indicating that between 286,844 and 430,267 samples out of the 1,434,224 possible combinations are suitable.

### 4.2. BCNB Synthetic Data Generation from BC-MLR

To evaluate the reproducibility of the proposed methodology, the same procedure described in the previous section was applied to breast cancer data. In this experiment, the reference data set is the Breast Cancer Machine Learning Repository (BC-MLR) while the incomplete data set is the Breast Cancer Core-Needle Biopsy (BCNB) data set. As presented in Section 3.1, both data sets share three common features. Given the relatively small size of both data sets, the goal is not only to generate synthetic features, but also to augment the number of samples. In addition, since the BCNB data set is missing the output variable, the intention is also to obtain this target value.

Unlike in Section 4.1, where a specific variable was generated, the goal here is to generate several synthetic features for the BCNB data set. The only condition to generate a new feature and add it to the known features in BCNB is that they must be present in the BC-MLR. The correlation between the BC-MLR variables and the target features class is considered to determine which features to generate.

 Table 2 shows the variables from BC-MLR and their corresponding correlations with the target attribute. The correlation between the known features is written in gray. Three other features with significant correlations with the target variable are chosen to be generated: node-caps, deg-malig, and irradiat.

**Figure 9:**
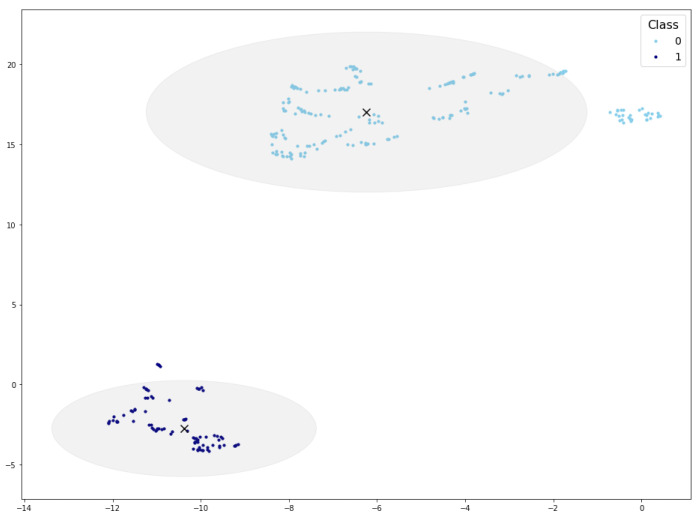
UMAP projection and cluster areas for the reference data in the first iteration. Cluster centroid marked with a black ×.

The procedure followed is the same as described in Section 4.1, but in this case it is applied iteratively, generating one feature at a time. In the first iteration, the reference data set consists of the three known attributes, and it is projected into the UMAP space, as shown in Figure 9. In subsequent iterations, the attribute to be generated is added to the reference data set, resulting in a new UMAP representation for each iteration. Each time the reference data change, the density threshold to define the cluster area should be adjusted; in this case, for all iterations the density threshold was set to 1. This iterative approach allows the generation of multiple features, each producing a distinct UMAP projection based on the updated reference data.

The generation of different features also involves defining the value of ϵ for the reliability score assignment. In this case, ϵ=1 was adopted for the three features.

In the data generation process, the reliability score threshold is set to $r_{c}=1$ for each iteration. The first feature to be generated is deg-malig, which can take a value of 1, 2, or 3, resulting in a total of 3×1058=3174 possible synthetic samples. Of these, 2558 samples were validated, accounting for 80.59% of the possible combinations.

The second generated feature is irradiat, which can take a binary value (`yes’ or `no’). Based on the previously generated data set containing 2558 samples, this step produces a data set of 5092 samples. Finally, the feature node-caps is generated, resulting in a final data set of 9427 samples.

By applying the proposed methodology iteratively, the initial incomplete data set with 1058 samples and three attributes is transformed into a partially synthetic data set with 8.9 times more samples (9427), six attributes, and the synthetic target feature (Class).

### 4.3. PI-CAI Validation

In order to validate the effectiveness of the algorithm, the ideal scenario would involve checking the actual attribute values of the incomplete data sets. For example, the patient_age values of the CIA database. However, this is not possible due to missing information. Therefore, the experimentation has been replicated for testing purposes by dividing the complete reference data set, PI-CAI, into two parts: PI-CAI 1 and PI-CAI 2.

To perform the validation, the complete and known PI-CAI database, with 1500 samples, was divided into five random groups, each containing 300 samples. One group was used as the reference data set (PI-CAI 1), while the other four groups served as incomplete data (PI-CAI 2). This process was repeated until all the data groups were utilized as reference data.

Using PI-CAI 1 as the reference data and PI-CAI 2 as the incomplete data, we applied our previously described methodology. By comparing the generated samples with the real data available in PI-CAI 2, we can evaluate the accuracy of the algorithm in imputing missing values and generating synthetic samples.

For each iteration, the methodology was applied, generating validated data. The validated data contained synthetic samples that did not correspond to any real patient and also correctly imputed data, by imputing the correct missing value of the incomplete data. After each iteration, the number of correctly imputed and synthetic generated data was recorded and averaged across the five iterations. This averaging process provides a more stable estimation of the performance of our algorithm.

**Figure 10:**
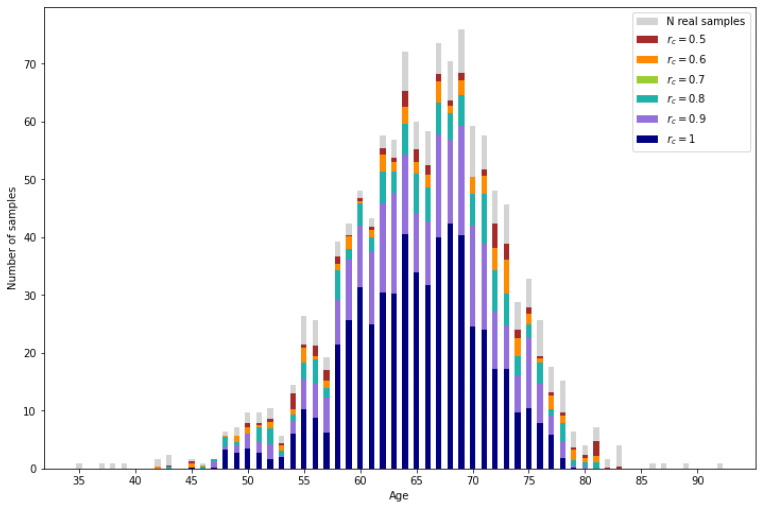
Real samples from the incomplete database (light gray) and correctly imputed samples for different reliability values.

In Figure 10, the amount of correctly imputed data is represented for each reliability threshold. As a reference, the number of samples originally corresponding to each age in the reference data set (PI-CAI 2) are represented in gray. Then, the number of correctly imputed samples per age are represented for the different reliability thresholds. For a more numerical interpretation, the percentage of correctly imputed data for certain age ranges is shown in Table 3. As expected, the number of correctly imputed samples increases as the reliability threshold decreases.

**Figure 11:**
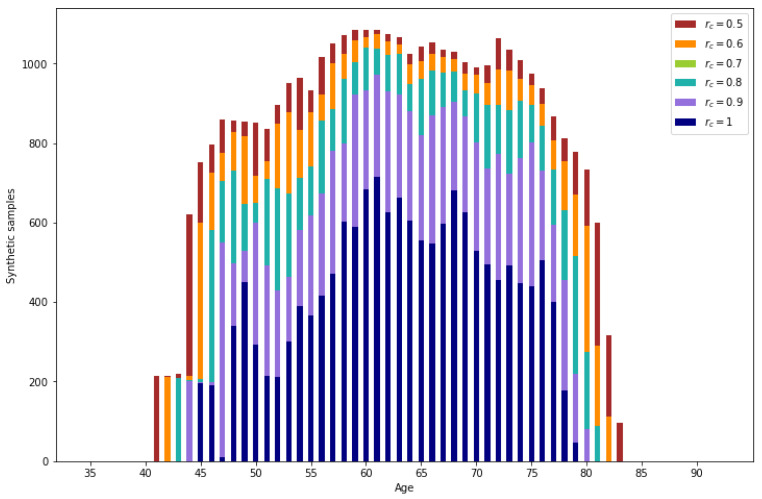
Number of synthetic samples per age.

All the validated samples that do not correspond to real data are synthetic samples. In Figure 11, the number of generated samples per age depending on the reliability threshold can be observed. It is observed that the number of samples is augmented significantly. Also, as expected, ages with lower representation result in a lower number of generated samples.

For lower reliability values, a higher number of generated data is obtained. From the generated data, a percentage between 3.49% ($r_{sc}=1$) and 2.79% ($r_{sc}=0.5$) corresponds to correctly imputed data, the rest of the generated data are synthetic samples.

**Figure 12:**
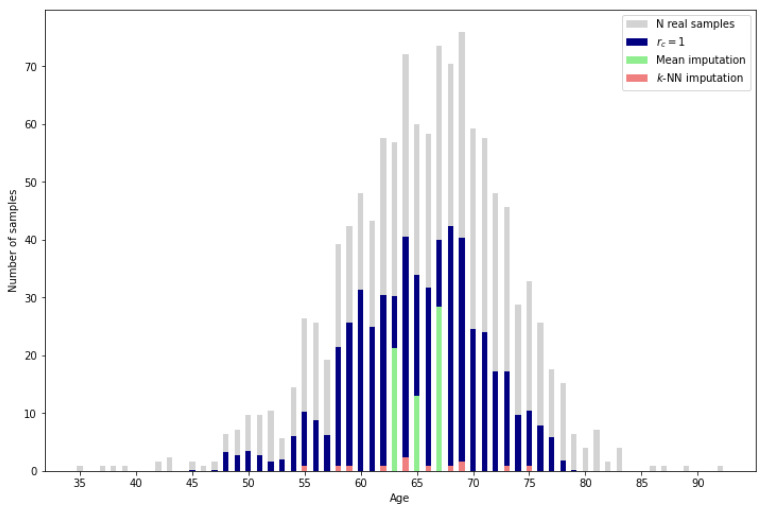
Imputation performance of mean and *k*-NN (k=10) vs. proposed methodology.

Aside from the usability of the synthetic samples, the proposed methodology presents a higher imputation performance than the typical methods such as mean or *k*-NN imputation. As can be observed in Figure 12, the mean imputation, which assigns the mean value of the reference data for the missing variable to all the missing samples, only obtains a low percentage of correct imputation for the resulting mean ages. On the other hand, *k*-NN imputation calculates the mean values of the missing variable based on the nearest neighbors, identified by computing the Euclidean distance between the feature vectors of the samples. It can be observed that there are more ages with correctly imputed samples than with mean imputation, but the amount of correctly imputed samples is very low. Finally, by applying the proposed methodology with the most restrictive reliability score $r_{sc}=1$, the number of correctly imputed samples increases significantly. A more detailed perspective can be obtained in Table 3 by comparing the exact percentage of correctly imputed samples.

Given that traditional methods lack the capability to leverage complete data as reference information, a direct comparison with the proposed methodology is not entirely appropriate. However, for a general sense of performance, it is worth noting that even the most restrictive reliability score in our method results in a validated sample percentage above 25% while the traditional methods only reach 0.45% and 2.02%.

Also, a possible criterion to determine the reliability score is to consider the percentage of correctly imputed samples from the generated data. It can be seen that the percentage does not change between $r_{c}=0.7$ and $r_{c}=0.8$. However, the largest drop in the percentage occurs between $r_{c}=0.9$ and $r_{c}=1$.

## 5. Discussion

The proposed methodology has demonstrated a robust capability to generate synthetic data, achieving an augmentation of between thirteen and thirty times (depending on the selected reliability threshold) the amount of incomplete data in the context of prostate cancer databases. In the case of breast cancer, the original data sets are smaller, yet the methodology successfully yields a partially synthetic data set that is approximately nine times larger than the original. Notably, this augmentation includes the generation of three attributes along with the target variable.

Moreover, for the validation against the PI-CAI data set, the percentage of correctly imputed values underscores the effectiveness of the proposed methodology, particularly when compared to traditional imputation techniques such as mean imputation or k-nearest neighbors (*k*-NN) imputation.

However, the methodology relies on a reference data set, which poses a potential limitation. In practical use cases, especially in clinical settings, the availability of a well-structured reference data set may not always be guaranteed. If such a data set is absent, the performance of the methodology could be compromised. To address this issue, alternative approaches such as using publicly available databases or synthetic reference data sets could be explored.

One key advantage of generating this large volume of data is that it allows for machine learning models’ generalization capabilities to be improved in low-data environments. The ability to generate high-quality synthetic data may reduce overfitting in models and allow for more robust decision making in clinical and other sensitive domains.

Yet, the advantage and disadvantage of generating a large amount of data is that, without knowing the expected value, it becomes challenging to differentiate between imputed and synthetic samples. This ambiguity benefits synthetic data generation, as all the data can be utilized without privacy concerns, given that the small percentage of real data cannot be distinguished.

However, this methodology may not be suitable for simple imputation, as the correctly imputed samples are mixed with synthetic data. Despite this, since multiple values are generated for each entry, the method could be adapted for multiple imputation. Each generated value could be assigned to the missing data in different data sets, creating multiple imputed data sets for analysis. Given that the number of assigned values for each entry is not constant, adjustments should be made to the number of iterations for multiple imputation.

While this study applied UMAP for dimensionality reduction on a data set with relatively low dimensionality, reducing a maximum of six dimensions to two, the full potential of UMAP in handling very high dimensional data remains to be explored. UMAP is particularly powerful in applications involving hundreds of dimensions, such as genomic or proteomic data sets.

In summary, while the methodology offers clear advantages in terms of synthetic data generation and augmentation, its dependency on reference data and its potential limitations in simple imputation tasks warrant further investigation. Nonetheless, its broad applicability in fields with available reference data sets makes it a valuable tool for data augmentation, particularly in healthcare.

## 6. Conclusions

The aim of this research was to develop a methodology for generating data from an incomplete database by leveraging a complete database as reference data, while addressing privacy concerns. The proposed methodology successfully imputed missing data and generated synthetic samples, surpassing the number of incomplete data entries.

The methodology provides a secure framework for data augmentation by utilizing data from different centers without the need to transfer sensitive information. Additionally, it yields superior results for data imputation tasks.

The developed methodology has practical applications in generating partially synthetic data that do not correspond to any specific patient. Also, as discussed throughout the paper, a partially synthetic data generation method can be adapted to produce fully synthetic data sets and imputed data sets. Consequently, the proposed method can be applied to various data generation scenarios. This has significant implications for data-driven research and decision making in healthcare and other fields.

A secondary aim of this article was to provide a formal definition of the synthetic data generation problem, specifically addressing cases of partially synthetic data generation. This objective has been successfully achieved, as the paper articulates a clear and formal definition of the problem, thereby contributing to the ongoing discourse in the field of data synthesis.

In conclusion, the proposed methodology represents a valuable tool for generating synthetic data, providing a balance between data utility and privacy preservation. Further research can explore its application in other domains and investigate additional methods for enhancing its performance and scalability. In particular, testing alternative dimensionality reduction methods across diverse data sets with varying characteristics and higher dimensionalities would provide valuable insights into its adaptability and effectiveness. Additionally, by refining the methodology for fully synthetic data generation, its outcomes can be more directly compared to established methods, facilitating a comprehensive evaluation of its potential across fields.

## Figures and Tables

**Table 1:** Reliability scores. Value ε depends on the range of the synthetic feature.

Feature Disparity ($f_{\mathrm{disp}}$)	Reliability Score ($r_{c}$)
$f_{\mathrm{disp}}\leq \varepsilon$	1
$\varepsilon <f_{\mathrm{disp}}\leq 2\varepsilon$	0.9
$2\varepsilon <f_{\mathrm{disp}}\leq 3\varepsilon$	0.8
$3\varepsilon <f_{\mathrm{disp}}\leq 4\varepsilon$	0.7
$4\varepsilon <f_{\mathrm{disp}}\leq 5\varepsilon$	0.6
$5\varepsilon <f_{\mathrm{disp}}\leq 6\varepsilon$	0.5

**Table 2:** Feature correlation with target variable. The correlation between the known features is written in gray. Significant feature correlations with the target variable are bolded.

Feature	Correlation
age	−0.072
menopause	0.052
tumor-size	0.13
inv-nodes	0.27
node-caps	**0.24**
deg-malig	**0.3**
breast	−0.059
breast-quad	0.037
irradiat	**0.19**

**Table 3:** Percentage of correctly imputed samples with the proposed methodology, mean and *k*-NN imputation.

Age Range		35–44	45–54	55–64	65–74	75–84	85–92	Mean %
UMAP PSDG	$r_{sc}=0.5$	16.67	83.93	91.73	88.09	66.26	0.00	57.78
	$r_{sc}=0.6$	11.11	78.57	89.03	85.11	59.09	0.00	53.82
	$r_{sc}=0.7$	5.56	70.24	84.99	80.19	52.97	0.00	48.99
	$r_{sc}=0.8$	5.56	70.24	84.99	80.19	52.97	0.00	48.99
	$r_{sc}=0.9$	0.00	54.46	76.25	71.30	41.61	0.00	40.60
	$r_{sc}=1.0$	0.00	33.04	53.39	48.68	22.73	0.00	26.31
Mean		0	0	4.93	7.17	0	0	2.02
*k*-NN		0	0	1.30	0.69	0.70	0	0.45

## Data Availability

The original data presented in the study are openly available in the provided repository links.

## Abbreviations

The following abbreviations are used in this manuscript:

VAEs	Variational Autoencoders
GANs	Generative Adversarial Networks
DM	Diffusion Models
BGM	Bayesian Gaussian Mixture
PATE	Private Aggregation of Teacher Ensembles
MCMC	Markov Chain Monte Carlo
LSTM	Long-Short Term Memory
MI	Multiple Imputation
MICE	Multivariate Imputation by Chained Equations
UMAP	Uniform Manifold Approximation and Projection
t-SNE	t-distributed stochastic neighbor embedding
PCA	Principal Component Analysis
EHR	Electronic Health Record
IoMT	Internet of Medical Things
PI-CAI	Prostate Imaging and Cancer Analysis Information
CIA	Cancer Imaging Archive
GS	Gleason Score
PSDG	Partially Synthetic Data Generation
PSA	Prostate-Specific Antigen
